# Colchicine for Coronary Artery Disease: A Review

**DOI:** 10.3389/fcvm.2022.892588

**Published:** 2022-06-16

**Authors:** Tao Chen, Guihong Liu, Bo Yu

**Affiliations:** ^1^Department of Cardiology, The First Affiliated Hospital of China Medical University, Shenyang, China; ^2^Department of Oncology, West China Hospital, Sichuan University, Chengdu, China

**Keywords:** colchicine, inflammation, coronary artery disease, percutaneous coronary intervention, coronary artery bypass grafting

## Abstract

Coronary artery disease is a serious threat to human health. More and more evidences indicate chronic inflammatory plays a key role in the development of this disease. Inflammation markers are gradually used in the diagnosis and treatment. Although the treatment of coronary heart disease with colchicine is still controversial, more and more studies showed that patients can benefit from this medicine. In this review, we discuss and summarize colchicine on essential pharmacology, anti-inflammatory mechanism of action, and the most important and recent clinical studies. According to these literatures, colchicine possibly will possibly become a new valuable and cheap medicine for the treatment of coronary artery disease.

## Introduction

**FIGURE 1:**
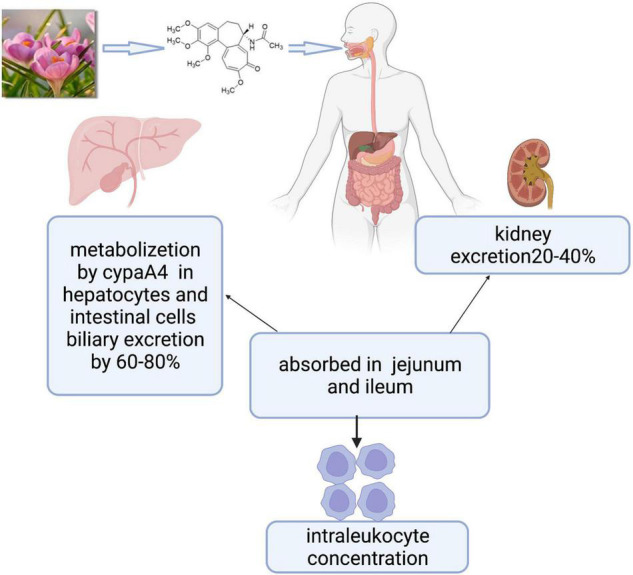
Colchicine is the active principle derived from colchicum autumnale plants whose formula is C22H25NO6, including three rings. Colchicine is absorbed in the jejunum and ileum, metabolized mainly by the CYP3A4 in the liver and intestine, and cleared by bile and kidney. Biological effects are mainly related to intraleukocyte concentrations.

Coronary artery disease (CAD) is a pathological process characterized by the accumulation of atherosclerotic plaques in epicardial arteries (1, 2). Lifestyle adjustment, drug therapy, and invasive therapy can change this process. Despite the availability of safe and effective low-density lipoprotein (LDL)-lowering therapy and antiplatelet therapy, the incidence of cardiovascular disease has been increasing (3). In recent viewpoint, arteriosclerosis is not only caused by lipid accumulation but also a chronic inflammatory reaction. Some studies showed that more than 50% of cardiovascular events may be attributed partly to inflammation (4–6). The recent consensus published by the European Society of Cardiology (ESC) confirmed that inflammation is an important factor in atherosclerosis (2). The CANTOS study was the first large-scale clinical study to confirm that anti-inflammatory treatment could reduce cardiovascular events, providing definite evidence for the inflammation hypothesis of arteriosclerosis with a reduction from baseline in interleukin (IL)-6 (Figure 1). But canakinumab is limited in the cardiovascular field because of its high price and causing serious infections (7). However, the CIRT study showed methotrexate did not decrease the level of IL-1β, IL-6, or C reactive protein (CRP) and did not reduce cardiovascular events (8). Colchicine, one of the oldest drugs, is widely used for acute gout, familial mediterranean fever, and pericarditis, Behcet’s disease (9). Over the past decades, with advances in the knowledge of cytoskeletal microtubules (MT) and anti-inflammatory effects of colchicine, low-dose colchicine (0.5–1.0 mg/daily) was increasingly administered for the therapy of cardiovascular diseases such as CAD, postoperative atrial fibrillation (in cardiac surgery), cardiac hypertrophy-associated heart failure (10). What’s more, in an animal experiment, colchicine improved left ventricular (LV) function in coxsackievirus 3 (CVB3) induced myocarditis and decreased cardiac and splenic family pyrin domain-containing protein 3 (NLRP3) inflammasome activity (11). A large number of studies about colchicine make progress in the treatment of CAD (Table 1). The aim of this review is to discuss pharmacology, anti-inflammatory mechanism of action, and recent clinical studies of colchicine in CAD.

**TABLE 1:** A summary of important clinical studies about colchicine in coronary artery disease.

Study	Study design	Sample size Colchicine/control	Clinical setting	Colchicine dose	Median follow up	Main results
**WITH GOUT**						
Solomon et al. (38)	Retrospective cohort study	1002 (501/501)	Patients with gout	—	16.5 months	Reduced incidence of composite of MI, stroke, TIA adjusted HR 0.51 (95% CI: 0.30–0.88) *P* = 0.016 and all-caused mortality (adjusted HR 0.27 95% CI: 0.17–0.43)
Crittenden et al. (39)	Retrospective cohort study	1288 (576/712)	Patients with gout	—	—	Reduced incidence MI (RR 0.46 *P* = 0.03) No significant change in mortality (*P* = 0.18)
**CCS**						
LoDoCo trial (40)	RCT	532 (282/250)	CCS	0.5 mg daily	36 months	Reduced incidence of CV death, non-cardioembolic stroke, ACS and out-of-hospital cardiac arrest (HR 0.33 95% C1: 0.18–0.59)
LoDoCo2 trial (41)	RCT	5522 (2762/2760)	CCS	0.5 mg daily	28.6 months	Reduced incidence of CV death, myocardial infarction, ischemic stroke, or ischemia-driven coronary revascularization (HR 0.69 95% CI: 0.57–0.83)
**ACS**						
Deftereos et al. (48)	RCT	151(77/74)	STMI	Loading dose of 2 mg and continuing with 0.5 mg twice daily	—	CK-MB AUC nearly half than the placebo group (*p* < 0.001). Smaller indexed CMR-defined MI size in the colchicine group (18.3 vs. 23.2 mL/1.73 m2, *p* = 0.019). Smaller relative MI size as a proportion of LV myocardial volume in the colchicine group (13.0% vs. 19.8%, *p* = 0.034).
COLCOT trial (49)	RCT	4745 (2366/2379)	MI (<1 month)	Colchicine 0.5 mg daily	22.6 months	Reduced incidence of CV death, cardiac arrest, myocardial infarction, stroke, or urgent hospitalizations for angina (HR 0.77 95% CI: 0.61–0.96)
COPS trial (50)	RCT	795 (396/399)	ACS	0.5 mg twice daily for the first month, then 0.5 mg daily for 11 months	12 months	The primary outcome of all-cause mortality, ACS, ischemia-driven urgent revascularization, and non-cardioembolic ischemic stroke did not differ between colchicine and placebo 24 vs. 38 events (*P* = 0.09)
**PCI**						
O’keefe et al. (51)	RCT	197 (130/67)	Patients undergoing PCI	0.6 mg twice daily	6 months	Sessions had restenosis to 47% lumen diameter narrowing in the placebo-treated group compared with 46% in the colchicine -treated group (*p* = NS)
Deftereos et al. (35)	RCT	196 (100/96)	Patients undergoing PCI	0.5 mg twice daily	6 months	Stent restenosis rate was 16% in the colchicine group compared with 33% in the control group (OR: 0.38, 95% CI: 0.18–0.79, *p* = 0.007)
COLCHICINE-PCI trail (52)	RCT	400 (206/194)	Patients undergoing PCI (50% ACS patients)	1.2 mg 1–2 h pre-procedure followed by 0.6 mg 1 h later or immediately preprocedure	30 days	The primary outcome of PCI-related myocardial injury did not differ between colchicine and placebo groups (57.3% vs. 64.2%, *P* = 0.19)

*MI, myocardial infarction; TIA, transient ischemic attack; HR, hazard ratio; CI, confidence interval; RR, relative risk; CCS, chronic coronary syndrome; RCT, randomized controlled trial; CV, cardiovascular; ACS, acute coronary syndrome; STEMI, ST-elevation myocardial infarction; AUC, area under curve; CMR, cardiovascular magnetic resonance; LV, left ventricle; PCI, percutaneous coronary intervention; NS, no significance; OR, odds ratio.*

We searched through electronic databases (MEDLINE/PubMed, EMBASE, and Google Scholar) using the search terms “colchicine” AND “cardiovascular disease” OR “coronary artery disease” OR “chronic coronary syndromes” OR “acute coronary syndromes” OR “percutaneous coronary intervention” OR “coronary artery bypass grafting.” The research was restricted to the English language.

## Pharmacology and Anti-Inflammatory Mechanism of Colchicine

### Pharmacology

Colchicine is an alkaloid with the formula C22H25NO6 (Figure 1), including three rings (12). The jejunum and ileum are the main sites of colchicine absorption. Following the oral route, peak plasma concentration occurs 1 h after administration, and the bioavailability can vary from 24 to 88% of the administered dose. The steady-state was reached at 8 days after the first oral administration, and the maximum anti-inflammatory effect was reached at 24–48 h. Colchicine and its metabolites are mainly cleared by kidney and bile and the elimination half-life ranged from 20 to 40 h. Pharmacokinetic/pharmacodynamic studies show that the biological effects of colchicine are related to intraleukocyte concentrations instead of plasma concentrations. Colchicine preferentially binds to three proteins including tubulin, cytochrome p3a4 (CYP3A4), and *P*-glycoprotein (13). The tubulin prevents persistently the fusion of autophagic vacuoles with lysosomes in neurons, bone marrow, and muscle, resulting in the risk of damage to these organ systems, especially in patients with liver and/or renal insufficiency. CYP3A4 and *P*-glycoprotein largely contribute to affecting colchicine metabolism and colchicine’s drug-drug interactions. CYP3A4 exists in hepatocytes and intestinal cells and metabolizes colchicine to 2- and 3-desmethylcolchicine. *P*-glycoprotein is an ATPase efflux pump, which exists in intestinal cells, liver cells, kidney cells, and the blood-brain barrier; it can affect the absorption of colchicine from the gastrointestinal tract. The prescribed dose, kidney or liver disease, and nature of adjunctive medication can trigger the risk of severe drug-drug interactions of colchicine. The combination with P-glycoprotein inhibitors (e.g., cyclosporine, calcium channel blockers, and ranolazine) or CYP3A4 inhibitors (e.g., clarithromycin, itraconazole, fluoxetine, ketoconazole, nefazodone, and cimetidine), makes colchicine metabolism disorders, further leads to concentration of colchicine increase, and finally happens drug poisoning (13, 14).

### Anti-inflammatory Mechanisms of Action

**FIGURE 2:**
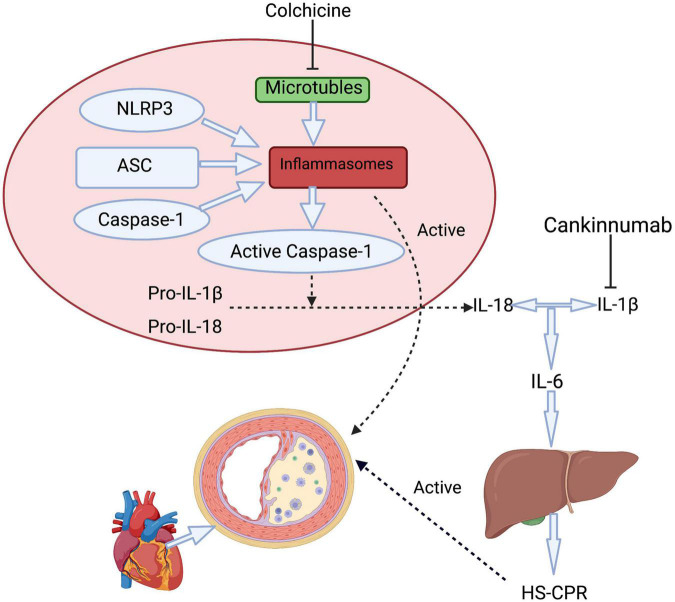
A simplified approach to inflammasome activation pathways includes therapy mechanisms of colchicine and canakinumab. Colchicine affects the NLRP3 inflammasome by inhibiting the function of microtubules, further resulting decrease in the downstream inflammatory factors include IL-1β, IL-6, and HS-CRP. Canakinumab prevents IL-1β and further reduces the release of IL-6 from various types of cells. hs- CRP is produced in hepatic through regulation by IL-6. ASC, apoptosis-associated speck-like protein containing a CARD; NLRP3, NLR family pyrin domain containing 3; IL, interleukin; hs- CRP, High-sensitivity C-reactive protein.

Colchicine was originally extracted from the autumn crocus as an anti-inflammatory drug and used by the ancient Greeks and Egyptians (15–17). The anti-inflammatory mechanism of colchicine mainly includes the following aspects. (1) Colchicine binds with high affinity to the specific domain of β-tubulin, resulting in (i) inhibition of tubulin assembly into MT, (ii) disassembly of preformed MT, and/or (iii) inhibition of membrane-bound tubulin sensitive cellular processes (10, 18), Through the mechanisms, different functions of MT are impaired such as amoeboid movements of leucocytes, exocytosis, and phagocytosis, separation of chromosome pairs during mitosis (19). Smooth muscle cells (SMC) secretion and other cellular atherogenic phenomena can be inhibited by colchicine through its anti-microtubule effect (18, 20), and multiple inflammatory factors are down-regulated by the effect of MT (17). (2) Colchicine can suppress NLRP3 inflammasome being present in myeloid cells (Figure 2), including monocytes, neutrophils, and eosinophils or its downstream inflammatory cytokine activation (21). Highly mature inflammatory cytokines IL-1β and IL-18 produced from NLRP3 lead to the progression of atherosclerotic plaques and even shedding of plaques (22, 23). In a large number of trials, NLRP3 expression and downstream cytokines in CAD were significantly higher compared with normal controls (24, 25). Blocking NLRP3 inflammasome not only inhibits oxidized LDL and cholesterol crystal-induced foam cell formation (26) but also has a good influence on ischemia/reperfusion injury, bringing about smaller infarct size and less fibrosis in mice (27). (3) High-sensitivity C-reactive protein (hs-CRP) as a downstream important effector of NLRP3 inflammasome is produced in hepatic through regulation by interleukin 6 (IL-6) (28). Colchicine can decrease significantly hs-CRP which is closely related to arteriosclerosis (29, 30). In the CANTOS study, the reduction of hs-CRP in the treatment group obviously increased cardiovascular benefits and reduced cardiovascular and total mortality (7). Nidorf et al. demonstrated that colchicine can effectively decrease hs-CRP in patients with chronic coronary syndrome (CCS) (31). Measurement of hs-CRP has been shown to independently predict future cardiovascular events in patients with CCS (32), and those with acute coronary syndrome (ACS) (33). (4) Expression of surface markers of platelet activity can be reduced and leukocyte-platelet aggregation can be inhibited by colchicine, which is a major contributor to atherothrombosis (34). Recent data showed that colchicine has antiplatelet activity (35, 36). (5) Colchicine treatment significantly reduces the expression of key inflammatory-related miRNA (37). (6) Colchicine also affects endothelial function (17). Colchicine improves endothelial function through its anti-inflammatory effect in CAD patients with leukocyte activation and reduces the adhesion of leukocytes to endothelium (16, 29). (7) In addition, the synthesis of prostaglandin E2, leukotriene B4, TNF-A, and TxA2, as well as the activity of COX-2 can be inhibited by Colchicine. Even at low doses, it inhibits inflammation by reducing the expression of *E* and *P*-selectin and weakens the adhesion of polymorphonuclear neutrophils (PMN) to the endothelium and inhibits neutrophil migration (16, 17).

## Research Progress of Colchicine in the Treatment of Coronary Artery Disease

### Chronic Coronary Syndromes

Chronic coronary syndrome is usually characterized by a reversible myocardial supply-demand mismatch. It is associated with ischemia or hypoxia, usually induced by exercise, emotion, or other stress, and occasionally occurs spontaneously (2). Despite routine treatment with antiplatelet and statins, patients are still at high risk for cardiovascular events. These treatments do not interfere with inflammation. A retrospective study matched 501 users of colchicine for gout with an equal number of non-users for a median follow-up of 16.5 months. Cardiovascular (CV) events occurred in 28 patients of 501 (5.6%) who received colchicine and 82 patients of 501 (16.4%) assigned no colchicine (hazard ratio [HR] 0.51, 95% confidence interval [CI] 0.30–0.88, *p* = 0.016) and a 73% reduction in all-cause mortality (HR 0.55, 95% CI: 0.35–0.85, *p* = 0.007) (38). Another retrospective study included 1288 gout patients, which were divided into Colchicine (*n* = 576) group and no colchicine (*n* = 712) group. Prevalence of (myocardial infarction) MI occurred in 1.2% of patients who received colchicine and 2.6% of patients assigned no colchicine (relative risk [RR]:0.46, *p* = 0.03). Fewer deaths and lower CRP levels appeared in the colchicine group, although without statistical significance (39). The LoDoCo trial showed that low-dose colchicine reduced the risk of plaque instability, thereby improving clinical outcomes in patients with CCS. The primary outcome was observed in 15 patients of 282 (5.3%) among users and 40 patients of 501 (16.0%) assigned among non-users (HR: 0.33; 95% CI: 0.18–0.59, *p* = 0.001). A low dose of colchicine in combination with other standard secondary prevention therapies occurred the prevention of cardiovascular events for patients with CCS (40). Recently, the LoDoCo2 study, published in the New England Journal, further confirmed the role of colchicine in the treatment of CCS, which matched 2762 users with 2760 non-users for a median of 28.6 months. The primary outcome which included cardiovascular death, spontaneous myocardial infarction, ischemic stroke, or ischemia-driven coronary revascularization occurred in 187 patients (5.3%) who received colchicine and 264 patients (9.6%) who did not receive colchicine (HR, 0.69; 95% CI: 0.57–0.83, *P* < 0.001). A key secondary endpoint event occurred in 115 patients (4.2%) who received colchicine and 157 patients (5.7%) assigned no colchicine (HR, 0.72; 95% CI: 0.57–0.92, *P* = 0.007). Secondary endpoints were tested in a hierarchical manner. Notably, the incidence of death from non-cardiovascular was higher in the colchicine group than placebo group (incidence, 0.7 vs. 0.5 events/100 person-years; HR, 1.51; 95% CI: 0.99–2.31) (41). Both LoDoCo and LoDoCo2 include a large number of patients, Colchicine can be an important supplement for secondary prevention of CCS. Some meta-analyses have shown that the reduction of inflammatory factors and plaque stabilization can be achieved by oral colchicine (42, 43). In general, many trials have confirmed the effectiveness of colchicine in patients with CCS. The ongoing DRC-04 study will demonstrate the effectiveness and safety of colchicine on patients with diabetes mellitus type 2 (T2DM) and CCS (Table 2).

**TABLE 2:** Ongoing clinical studies of colchicine in coronary artery disease.

Study	NCT No.	Study design	Clinical condition	Target number	Interventions	Primary outcome measures
**CCS**						
DRC-04	NCT03376698	RCT, Phase 2	T2DM with CAD	69	Colchicine 0.5 or 0.25 mg daily vs. placebo	Change in serum hs-CRP
**ACS**						
COACS	NCT01906749	RCT, Phase 4	ACS	500	Colchicine 0.5 mg daily vs. placebo	Overall mortality, new acute coronary syndrome, and ischemic stroke
COCOMO-ACS	ANZCTR reg no: ACTRN1261800 0809235	RCT, Phase 2	ACS	82	Colchicine 0.5 mg daily vs. placebo	Coronary plaque minimum brous cap thickness on OCT at 12 months
COLCARDIO-ACS	ANZCTR reg no: ACTRN1261600 0400460	RCT, Phase 3	ACS with hs-CRP 2 mg/L	300	Colchicine 0.5 mg daily vs. placebo	Composite MACE (CV death, ACS, urgent revascularisation, non- fatal stroke) at a median follow-up of 3 years
Effect of Colchicine in Patients With Myocardial Infarction	NCT04218786	RCT, Phase 2	STEMI	800	Colchicine 0.5mg daily vs. placebo	Cardiovascular death Non-fatal myocardial infarction Resuscitated cardiac arrest Hospitalization for unstable angina
CADENCE	NCT04181996	RCT, Phase 3	T2DM or pre-diabetes and a recent Non-STEMI, STEMI, stroke or TIA	115	Colchicine 0.6mg daily vs. placebo	6 month change in FDG uptake TBR in the MDS
COLD-MI	NCT04420624	RCT, Phase2,3	MI	54	Colchicine 1 or 0.5 mg daily vs. placebo	Percentage of myocardial denervation
COLOCT	NCT04848857	RCT, Phase 4	ACS	128	Colchicine 0.5 mg daily vs. placebo	Changes of the thickness of fibrous cap of coronary artery plaque measured by OCT
CLEAR SYNERGY Neutrophil Substudy	NCT03874338	A sub study of multi center 2 × 2 randomized placebo controlled trial	STEMI	670	Colchicine vs. placebo	Change in soluble L-selectin between baseline and 3 months after STEMI
Post-MI PET Scan Imaging of Inflammation	NCT02281305	RCT, Phase 4	MI	20	Colchicine 2 mg loading dose; 0.5 mg bid for 5 days vs. placebo	Degree of inflammation of the involved myocardium as assessed by the PET scan
**PCI**						
COVERT-MI	NCT03156816	RCT, Phase 2	STEMI with PCI	194	Bolus colchicine 2 mg then 0.5 mg BD for 5 days vs. placebo	Infarct size (in% of LV mass) on CMR
CLEAR SYNERGY	NCT03048825	RCT, 2 × 2 factorial (with Spironolactone)	MI with PCI	7000	Colchicine 1 mg daily or spironolactone 25 mg daily vs. placebo	Composite of MACE (death, recurrent target vessel MI, stroke, or ischemia driven target vessel revascularization) Composite of cardiovascular death, recurrent myocardial infarction, or stroke Composite of cardiovascular death or new or worsening heart failure
ORCA	NCT04382443	RCT, Phase 4	PCI	450	BMS + Colchicine (0.5 mg twice a day during the first 3 months after stent implantation) vs. DES	Composite of death, MI and ischemic TVR death included cardiac, non- cardiac and non- determined
Colchicine in Periprocedural Myocardial Infarction: the Role of Alpha Defensin	NCT03735134	Non-Randomized. Not Applicable	MI with PCI	180	Colchicine vs. placebo	Occurrence of periprocedural myocardial infarction post elective PCI
Anti-inflammatory Effects of Colchicine in PCI (a substudy of the COLCHICINE-PCI trial)	NCT01709981	RCT, Phase 4	PCI	280	1.2 mg colchicine 1–2 h prior PCI, followed by 0.6 mg 1 hour later vs. placebo	Percent change in post-procedural IL-6 concentration from baseline to 30 min –1 h after PCI
**cerebrovascular disease and PAD**						
CONVINCE	NCT02898610	RCT, Phase 3	Ischemic attack, Transient stroke	2623	Colchicine 0.5 mg daily vs. placebo	Recurrence of non-fatal ischemic stroke (Any recurrence of non-fatal ischemic stroke) On-fatal major cardiac event (Non-fatal hospitalization for unstable angina, myocardial infarction, cardiac arrest) Vascular death (Fatal ischemic stroke, myocardial infarction, cardiac arrest)
LEADER-PAD	NCT04774159	RCT, Phase 3	Peripheral arterial disease	150	Colchicine 0.5 mg daily vs. placebo	Efficacy outcomes:major adverse cardiovascular and limb events Safety outcomes:gastrointestinal toxicity that leads to treatment interruption or discontinuation, infection (e.g., pneumonia) and incident cancer

*RCT, randomized controlled trial; T2DM, diabetes mellitus type 2; CAD, coronary artery disease; hs-CRP, high-sensitivity C-reactive protein; ACS, acute coronary syndrome; OCT, optical coherence tomography; CV, cardiovascular; MACE, major adverse cardiac events; STEMI, ST-elevation myocardial infarction; non-STEMI, non-ST-elevation myocardial infarction; TIA, transient ischemic attack; FDG, fluorodeoxyglucose; TBR, tissue to blood ratio; MDS, maximum disease segment; MI, myocardial infarction; PET, positron emission tomography; CMR, cardiovascular magnetic resonance; PCI, percutaneous coronary intervention; BMS, bare metal stents; DES, drug eluting stent; TVR, target vessel revascularization; IL-6, interleukin-6.*

### Acute Coronary Syndrome

Acute coronary syndrome was defined as symptoms of acute myocardial ischemia associated with either elevated troponin or ECG changes. The current treatments for ACS mainly include antiplatelet, anticoagulation, blood lipid regulation, and operations, such as percutaneous coronary intervention (PCI) or coronary artery bypass grafting (CABG) (44, 45). Even with the above treatments, mortality and morbidity in patients with ACS still keep increasing. Inflammation is involved in all stages of atherosclerosis, including plaque formation, progression, instability, and rupture. Anti-inflammatory treatment may be an effective breakthrough for ACS. In an animal trial, Colchicine can significantly reduce infarct area and cardiac fibrosis in mice, and improve hemodynamic parameters (46). Another animal trial showed the anti-inflammatory mechanism of colchicine can protect the myocardium in the rat MI model (47). In the above studies, a unique dose of colchicine by limitation of the inflammation at the early stage of MI decreased myocardial injuries, which was manifested as a reduction in infarct size and T troponin level at 24 h. More importantly, colchicine improved long-term cardiac remodeling by improving hemodynamic parameters and reducing myocardial fibrosis (46, 47). These animal studies provide some evidences for colchicine treatment of ACS. Deftereos et al. have demonstrated in a double-blind that a 5-day course of treatment combined with colchicine and PCI led to a reduction in infarct size in 151 patients with STEMI (48). In this trial, Colchicine significantly decreased circulating levels of CK-MB, troponin measured 72 h post-MI, and infarction volume measured using cardiac MRI compared to placebo (48). The COLCOT clinical trial including 4745 patients followed for a median of 22.6 months. The primary endpoint including death from cardiovascular causes, resuscitated cardiac arrest, myocardial infarction, stroke, or urgent hospitalization for angina leading to coronary revascularization occurred in 5.5% of the patients who received colchicine, as compared with 7.1% in the placebo group (HR, 0.77; 95% CI: 0.61–0.96, *P* = 0.02). The hazard ratios were 0.84 (95% CI: 0.46–1.52) for death from cardiovascular causes, 0.83 (95% CI: 0.25–2.73) for resuscitated cardiac arrest, 0.91 (95% CI: 0.68–1.21) for myocardial infarction, 0.26 (95% CI: 0.10–0.70) for stroke, and 0.50 (95% CI: 0.31–0.81) for urgent hospitalization for angina leading to coronary revascularization. The result showed the primary end point, stroke, urgent hospitalization for angina leading to coronary revascularization was significant lower in the colchicine group compared with the placebo group (49). The COPS randomized clinical trial recruited a total of 795 patients with ACS, 396 in colchicine group and 399 in the placebo group at 1 year of follow-up. The result showed that standard treatment with colchicine had no significant effect on cardiovascular outcomes and occurred a higher rate of mortality, such as 24 events in the colchicine group and 38 events in the placebo group (*P* = 0.09, log-rank). The rate of total death in the colchicine group was higher (8 versus 1; *P* = 0.017 log-rank), particularly, non-cardiovascular death in the colchicine group (5 versus 0; *P* = 0.024 log-rank). However, if the composite endpoint used only cardiovascular death rather than total death (cardiovascular death, stroke, ACS, and urgent revascularization) over the 12-month follow-up, the group of colchicine had a lower event rate (5.0% versus 9.5%; HR, 0.51 [95% CI: 0.29–0.89], *P* = 0.019). What’s more, at 400 days, there was a statistically significant difference between groups for the primary outcome (ACS, stroke, death, and urgent revascularization), with 24 events in the colchicine group compared with 41 events in the placebo group (*P* = 0.047, log-rank test) (50). Although COPS had negative results during 12 months of follow-up, we need to take into account the limitations of this trial, such as the insufficient sample size, low proportion of women, and limited follow-up time. When making a further analysis, firstly, the dose of colchicine was high in the COPS trial during the first month, and negative results may be caused by this factor. Secondly, the primary outcome at 12 months and 400 days were contradictory. It cannot be ruled out that the benefit further increases as the duration of colchicine administration is prolonged. Therefore, colchicine in the treatment of ACS is worth long-term observation. Although colchicine does not get consistent results in the treatment of ACS, many trials have been added to confirm the treatment of ACS with colchicine. We will see the results of more clinical studies about ACS in the future, including COACS, COCOMO-ACS, COLCARDIO-ACS, CADENCE, COLD-MI, COLOCT, etc.

### Percutaneous Coronary Intervention and Coronary Artery Bypass Grafting

Percutaneous coronary intervention is the most important way to treat CAD. The processes of PCI including wire injury, microdissections at the site of balloon inflations, and vascular trauma due to high-pressure balloon inflations can further aggravate plaque instability and inflammation (2, 44). Therefore, many scholars take special attention to the curative effect of controlling inflammation on PCI patients. Early studies on colchicine mainly focused on stenosis after PCI. The first study on the use of colchicine in PCI patients was published in 1992, including 197 patents, which found that colchicine 0.6 mg BD did not reduce vascular stenosis (51). A prospective, randomized clinical trial published in 2013 included 196 diabetic patients who received bare-metal stents and proved patients who received a 6-month course of 0.5 mg BD with colchicine can benefit. The angiographic in-stent restenosis rate occurred in 16% of the patients who received colchicine and 33% of patients assigned no colchicine (odds ratio [OR]: 0.38, 95% CI: 0.18–0.79, *p* = 0.007) (35). COLCHICINE-PCI randomized clinical trial recruited a total of 400 patients with 206 assigned to the colchicine group and 194 to the placebo group. The colchicine group was scheduled for acute preprocedural oral administration of colchicine, and the other group received an oral placebo. The composite outcome of death, non-fatal myocardial infarction, and target vessel revascularization at 30 days (11.7% versus 12.9%, *P* = 0.82), and the outcome of PCI-related MI (2.9% versus 4.7%, *P* = 0.49) did not differ between colchicine and placebo groups (52). There is very little information on the use of colchicine in CABG. Giannopoulos et al. demonstrated in a prospective, double-blind trial about CABG including 59 patients with 30 assigned to the colchicine group and 29 to the placebo group that treatment with colchicine led to a reduction in increases of hsTnT and CK-MB (53). In the trial, the median area under curve (AUC) for hsTnT was 20,363 pg h/ml (13,891–31,661) in the colchicine group versus 40,755 pg h/ml (20,868–79,176) in controls (*p* = 0.002). AUC for CK-MB was 1,586 ng h/ml (1,159–2,073) in the and colchicine group 2,552 ng h/ml (1,564–4,791) in control (*p* = 0.003). In general, a short perioperative colchicine administration can reduce the elevation of hsTnT and CK-MB compared with placebo (53). The result was partly attributed to the anti-inflammation of colchicine. At present, considering the number of trials and patients involved were too small, and the results were inconsistent, more trials are needed to take part. Ongoing clinical trials of colchicine in PCI include COVERT-MI, CLEAR-SYNERGY, ORCA, etc.

## Safety and Adverse Reactions

0.5–1.0 mg of colchicine per day has proven relatively safe, as evidenced in many studies. However, it is noteworthy that patients with severe kidney or hepatic disease have been expelled from most of these trials. If patients have severe renal or liver function damage, the safety of colchicine decreases and even causes fatal toxicity. The therapeutic window of colchicine is narrow (54). Colchicine is toxic at doses greater than 0.1 mg/kg and lethal at 0.8 mg/kg (13). A meta-analysis focusing on adverse events on colchicine for the treatment of cardiovascular diseases demonstrated the occurrence of adverse events with colchicine was reported in 15.3 vs. 13.9% of patients [RR 1.26, 95% CI: 0.96–1.64, *P* = 0.09] (55). However, most patients can tolerate a low dose of colchicine well over the long term. The most common colchicine-related adverse events are gastrointestinal discomforts including nausea, vomiting, diarrhea, and abdominal discomfort. Other adverse events include myotoxicity, hepatic event, hematology event, cutaneous event, and infectious event (54). In particular, colchicine may increase the incidence of pneumonia. In COLCOT trial, Pneumonia as a serious adverse event occurred in 0.9% of the patients who received colchicine and 0.4% of patients assigned no colchicine (*P* = 0.03) (49). In another study, which included 24,410 patients, the overall incidence rates of pneumonia in the colchicine group were 18.6 per 1,000 person-years as compared with 12.6 per 1,000 person-years in non-colchicine group. Pneumonia was higher in the colchicine group [adjusted HR, 1.42; 95% CI: 1.32–1.53; *P* < 0.05]. The risk of pneumonia is also closely related to the cumulative dose and duration of use (56). Increasing the rate of infection may be attributed to the suppression of immunity. In addition, the increase in non-cardiovascular death also deserves attention. The LoDoCo2, COPS have mentioned that colchicine may increase the rate of non-cardiovascular death. Attention should also be paid to the combination of colchicine and other drugs such as CYP3A4 inhibitors, *P*-glycoprotein inhibitors, or statins. When combined with drug CYP3A4 inhibitors or *P*-glycoprotein inhibitors, the concentration of colchicine is increased, which leads to an increase in adverse events. Combination use of statins increases with may increase the risk of myopathy (54). Although colchicine is relatively safe, considering its narrow therapeutic range and interactions with other drugs, we need to pay attention to its adverse reactions, especially in patients with impaired liver and kidney function. When considering the symptoms of an adverse reaction, the clinician first confirms whether the adverse reaction is caused by colchicine. Blood concentration monitoring can provide more scientific clues. Once established, dosage reduction or withdrawal should be actively considered.

## Discussion

At present, Anti-inflammatory treatment for CAD is getting more and more attention but there are also many controversies. This article will discuss the following aspects. Firstly, the author considers it is highly possible that colchicine in the treatment of CAD will be written into guidelines, but it is too early to draw a conclusion at present. The results of five trials (LoDoCo, LoDoCo2, Deftereos et al., COLCOT, Spyridon Deftereos et al.) were positive and two trials [JAMES H. O’KEEFE, et al., COLCHICINE-PCI trail] were negative. The result of the COPS trial was special, with a negative result at 12 months of follow-up and a positive result at 400 days of follow-up. When we further analyze, the weight of positive results is larger than negative. A meta-analysis including LoDoCo2, COPS, COLCOT, Spyridon Deftereo et al., and LoDoCo trials indicated that the risk of major adverse cardiovascular events (MACE) can be reduced by low-dose colchicine (57). Furtherly analyzing the grouping situation, the results of colchicine in CCS are relatively consistent. However, the results of ACS and PCI with colchicine are not consistent. Many reasons should be considered for this phenomenon. To begin with, compared with CCS, the treatment process of ACS and PCI is more complex, with higher risk and worse prognosis. What is more, many factors affect the prognosis of ACS and PCI, including the condition of patients, timeliness of the visit, quality of medication administration, and quality of the surgeon. Therefore, the weight of anti-inflammatory therapy in the whole process is relatively small, which may be an important reason for the inconsistent outcome. Because of these reasons, enthusiasm for colchicine in the treatment of ACS and PCI is growing. Secondly, inflammatory markers for assessing and treating CAD are unclear, unlike blood lipids, especially low-density lipoproteins, which can be used as indicators for monitoring the effectiveness of statins. Although many inflammatory indicators have been mentioned, such as hs-CRP, IL-1β, IL-18, and IL-6. White blood cell was even considered to be an independent predictor of MACE in a prospective study (58). But the sensitivity and specificity of these inflammation indicators are controversial. Among the inflammation biomarkers, hs-CRP is the most widely studied. In CANTOS study, the reduction of hs-CPR is parallel to the reduction in cardiovascular events (7). However, in COLCHICINE-PCI trial, colchicine could prevent a rise of hs-CRP during an acute injury, but which could not reduce PCI-related myocardial injury or 30-day major cardiovascular events (52). In the small subgroup of COLCOT trial, a large (>65%) reduction in the C-reactive protein level occurred over the first 6 months after myocardial infarction in colchicine and control groups, but the difference between the changes in the groups was not significant (49). At present, routine assessment of hs-CRP levels does not be advised as part of risk assessment for cardiovascular prevention by the European Society of Cardiology guidelines (59), while the American College of Cardiology guidelines suggest aggressive testing for hs-CRP levels (60). Inflammation indicators need more data to confirm. Finally, based on the common etiology and pathophysiological grounds of arteriosclerotic diseases, whether colchicine can prevent and treat cerebrovascular disease and peripheral arterial disease (PAD) is also worth looking forward to. A systematic review and meta-analysis including four randomized controlled trials (RCTs) and one retrospective cohort study confirmed that patients with stroke may benefit from colchicine (61). Unfortunately, clinical trials of colchicine for peripheral artery disease are few. The Ongoing CONVINCE study with high-quality randomized data will prove colchicine for secondary prevention after stroke is effective and safety (62). An ongoing LEADER-PAD study will provide some information on PAD. At present, many results of colchicine for CAD are positive and significant. The official recommendation of colchicine for CAD by cardiovascular guidelines in the future is worth anticipating. What’s more, the effect of colchicine on cerebrovascular diseases and PAD is hopeful.

## Conclusion

Due to the high morbidity and mortality of CAD, new approaches are urgently needed. Current evidences show that inflammation is a key component in the pathogenesis of atherosclerosis. Inflammation indicators such as IL-6 and hs-CRP have been paid much attention to. Anti-inflammatory therapy may become one of the effective methods for the treatment of CAD. Colchicine effectively reduces the incidence of cardiovascular events through an anti-inflammatory mechanism. What is more, colchicine is cheap and relatively safe. At the same time, we need to care about the adverse reactions and drug interactions, in particular, colchicine may be associated with increased rates of pneumonia and non-cardiovascular death. It is highly probable that the inhibition of inflammation will become the important cornerstone of CAD treatment.

## Author Contributions

All authors listed have made a substantial, direct, and intellectual contribution to the work, and approved it for publication.

## Conflict of Interest

The authors declare that the research was conducted in the absence of any commercial or financial relationships that could be construed as a potential conflict of interest.

## Publisher’s Note

All claims expressed in this article are solely those of the authors and do not necessarily represent those of their affiliated organizations, or those of the publisher, the editors and the reviewers. Any product that may be evaluated in this article, or claim that may be made by its manufacturer, is not guaranteed or endorsed by the publisher.
